# The Key Role of Epigenetics in the Persistence of Asexual Lineages

**DOI:** 10.1155/2012/534289

**Published:** 2012-02-14

**Authors:** Emilie Castonguay, Bernard Angers

**Affiliations:** ^1^Wellcome Trust Centre for Cell Biology, University of Edinburgh, Mayfield Road, Edinburgh EH9 3JR, UK; ^2^Département de Sciences Biologiques, Université de Montréal, C.P. 6128, succursale Centre-ville, Montréal, QC, Canada H3C 3J7

## Abstract

Asexual organisms, often perceived as evolutionary dead ends, can be long-lived and geographically widespread. We propose that epigenetic mechanisms could play a crucial role in the evolutionary persistence of these lineages. Genetically identical organisms could rely on phenotypic plasticity to face environmental variation. Epigenetic modifications could be the molecular mechanism enabling such phenotypic plasticity; they can be influenced by the environment and act at shorter timescales than mutation. Recent work on the asexual vertebrate *Chrosomus eos-neogaeus* (Pisces: Cyprinidae) provides broad insights into the contribution of epigenetics in genetically identical individuals. We discuss the extension of these results to other asexual organisms, in particular those resulting from interspecific hybridizations. We finally develop on the evolutionary relevance of epigenetic variation in the context of heritability.

## 1. Introduction

Despite its increased cost relative to asexual reproduction, sexual reproduction is common in multicellular organisms, which can lead to the interpretation that there is an advantage to reproducing sexually. This topic has been the subject of much debate, and, in the last decades, several hypotheses have been proposed to explain why sexual reproduction is maintained in populations. These hypotheses generally can be divided into two classes: (i) sex creates the genetic diversity necessary to cope with environmental variation (Fisher-Muller accelerated evolution theory [1, 2]; Red Queen hypothesis [3]; Tangled bank hypothesis [4]) and (ii) sex allows purging of deleterious mutations [2, 5, 6]. These hypotheses are all based on the assumption that asexual lineages are evolutionary dead ends.

Asexual reproduction is the primary form of reproduction in bacteria, archaea, and protists. It is also not uncommon in multicellular eukaryotes and is found in many phyla, particularly in plants, arthropods, nematodes, and rotifers [7]. In plants and animals, obligate asexuality is a derived character. It often results from the hybridization of two individuals from different sexual species [8–10], producing fertile hybrids no longer capable of reproducing sexually.

Over half the taxa examined by Neiman et al. [10] were represented by asexual lineages estimated to be >500,000 years old. Notably, amongst the oldest asexual lineages are the bdelloid rotifers, reported to have evolved for tens of millions of years without sexual reproduction [11]. These examples constitute a serious challenge to the common view that asexuality increases long-term extinction rate.

Because they generally lack recombination and the possibility to create genetic variation in their offspring, asexual lineages are thought to be limited in their capacity to colonize new environments and respond to environmental fluctuations. However, several asexual lineages have been found to possess a large geographical distribution [7, 12–18]. To explain this observation, based on concepts of the General Purpose Genotype model [19], evolutionary persistent asexual lineages have been hypothesized to be generalists characterized by flexible genotypes that allow them to occupy wide ecological niches [12].

Under this model, asexual lineages would possess an important capacity for phenotypic variation. Genetic mutation and epigenetic modifications are molecular mechanisms known to sustain phenotypic variation (reviewed in [20]). Could these mechanisms explain the persistence of these “evolutionary scandals” [21]? As we will explain, this depends largely on the timescale at which they act.

Mutations are long-term acting mechanisms that can create phenotypic variation. Yet many asexual taxa are thought to be particularly efficient in DNA repair, which would allow them to reduce the accumulation of deleterious mutations. There is evidence for this in asexual taxa such as asexual weevils [22], aphids [23], darwinulid ostracods [24], *Daphnia* [25], and oribatid mites [26]. However, the oldest known asexual lineage, the bdelloid rotifers, displays higher accumulation of mutations than related sexual species [27]. While efficient DNA repair will reduce the load of deleterious mutations in asexual populations, they will consequently also possess less genetic diversity to face environmental variation. Therefore, whether this mechanism is prevalent or not, it cannot explain on its own the persistence of asexual lineages since it does not account for how they can respond to environmental variation.

How do asexual organisms face environmental variation without sexual recombination? In bdelloid rotifers, two alleles at a given locus will diverge over time due to their independent accumulation of mutations and lack of recombination, effectively resulting in two genomes within one organism (Meselson effect [11]). However, besides the bdelloid rotifers [11], the Meloidogyne root knot nematodes [28], and Holbøll's rockcress [29], most asexual lineages are not characterized by the Meselson effect [26, 30]. In some asexual lineages, this could be due to the counteracting effect of homogenizing mechanisms such as efficient DNA repair. Alternatively, these other lineages could simply still be too young for mutations to be accumulated.

It appears therefore that many asexuals do not possess any specific mechanism for generating genetic variation. Despite this, these lineages have faced environmental variation for several thousands to millions of years. Even organisms where the Meselson effect is observed have most likely not strictly relied on genetic variation to face environmental variability, as this mechanism is not expected to produce genetic variation at a timescale short enough to be relevant to that at which environmental perturbations occur.

Asexual lineages must therefore possess shorter-term acting mechanisms to face environmental variation. In the absence of genetic diversity, the ability of these organisms to respond to environmental variability will depend on their capacity for phenotypic plasticity ([31] and references therein).

Epigenetic modifications could be a shorter-term acting mechanism allowing the creation of phenotypic variation among genetically identical individuals [32–37]. Epigenetics refers to changes in gene expression stably propagated through cellular divisions that occur without changes in the DNA sequence but through, for example, chemical modifications to the DNA (e.g., DNA methylation) and its associated proteins, the histones [38]. DNA methylation, in particular, is the most studied epigenetic modification. Epigenetic modifications are stably inherited through cell divisions and can underlie phenotypic change at least throughout the lifetime of an individual. The phenotypic differences induced by epigenetic changes can create differences in individual fitness (e.g., [39, 40]). Specific environmental conditions have been shown to induce changes in epigenetic states (e.g., [37, 41–47]). Therefore, epigenetic modifications, unlike mutations, allow the genome to integrate extrinsic environmental signals. Importantly, DNA-methylation-driven phenotypic variation has also been observed to be transmitted across organismal generations [44, 48, 49].

In asexual organisms, epigenetic modifications could cause phenotypic differences among individuals that would affect a single generation of organisms or in some cases that could persist in asexually produced offspring. In the present discussion of asexual organisms, the concept of phenotypic plasticity will be used to describe phenotypic effects of epigenetic modifications affecting a single organismal generation. However, in some other papers, the concept has been expanded to include both single-generation and transgenerational epigenetic modifications (see [33, 35, 50] for further discussion on the relationship between epigenetics and phenotypic plasticity).

Epigenetic modifications might be an important mechanism for creating phenotypic variability in asexual organisms, allowing them to face environmental variability [34, 36, 37]. The role of epigenetics could be especially important in the earlier stages of the existence of asexual lineages, when the effect of longer-acting mechanisms such as mutation is not yet felt. Indeed, epimutations occur at a greater rate than mutations [51–53], and, consequently, epigenetic variation among individuals is likely to precede genetic variation. Also, like mutations, epimutations are not all advantageous, but disadvantageous epimutations have the advantage of being reversible.

Some evidence for the role of epigenetics in asexual organisms comes from studies of asexual dandelions where variation in DNA methylation was detected among individuals of a single apomictic lineage [36, 37]. This variation was transmitted across generations and was sequence independent (see [33, 54] for discussion on the evolutionary significance of different degrees of dependence of epigenetic variation on genetic variation). Moreover, various stresses were shown to induce inheritable variation in DNA methylation [37]. Our group's recent work on the asexual fish *Chrosomus eos-neogaeus* [55] represents to our knowledge the first investigation of variation in DNA methylation associated with the environment in a naturally occurring asexual animal lineage. In the following paragraphs, we will discuss the ways by which epigenetic variation can play a role in the evolutionary success of asexual lineages in light of our results on *C. eos-neogaeus*.

## 2. Phenotypic Variation in Asexual *Chrosomuseos-neogaeus* Hybrids

**Figure 1:**
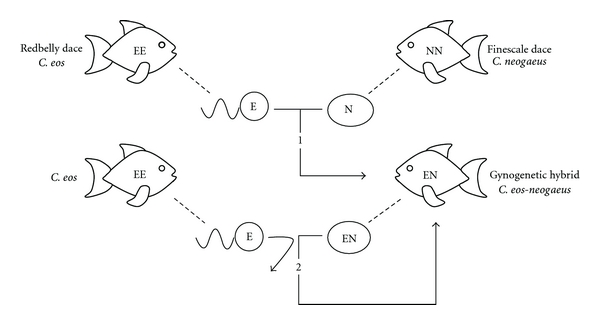
Expected mechanism leading to the natural occurrence of asexual hybrids in *Chrosomus eos-neogaeus.* (1) Gynogenetic hybrids resulted from hybridizations between female *Chrosomus neogaeus *and male *C. eos.* All-female hybrids are composed of one haploid set of chromosomes from each parental species. (2) Asexual reproduction occurs via gynogenesis: the entire genomic constitution of the mother is transmitted to the eggs and sperm from parental species is required only to initiate cleavage. The resulting offspring are genetically identical to the mother.

Vertebrates are ancestrally sexual and all known (obligate) asexual vertebrates have arisen from hybridizations. Asexual *Chrosomus eos-neogaeus* result from hybridizations between the northern redbelly dace *Chrosomus eos* and the finescale dace *Chrosomus neogaeus* (Pisces: Cyprinidae) (Figure 1). These all-female hybrids produce unreduced eggs without recombination [56, 57]. They are gynogens so the sperm from one of the two parental species is required to activate embryogenesis, but the paternal genome is not incorporated into the egg. The resulting offspring are diploid individuals genetically identical to each other and to their mother [56, 58].

While parental species and hybrids are common and widely distributed through the northern part of North America, only a limited number of different asexual lineages have been detected [59]. The hybridization events that gave rise to *C. eos-neogaeus* hybrids took place in glacial refuges during the Pleistocene. At the end of the glaciation, the hybrids dispersed throughout North America [59]. The same lineage could therefore occur in different types of environments. This diversity in habitat use of a single diploid clonal lineage has indeed been documented [60, 61].

*Chrosomus eos-neogaeus* populations appear to possess no interindividual genetic variation. Indeed, in several lakes where these hybrids are found, a single clonal lineage is present and only a few lineages have been detected in every region studied so far [56, 59, 61–63].

A single *C. eos-neogaeus* lineage could therefore be found across a broad geographical and ecological range, indicating the capacity of these asexual organisms to face environmental variability. A number of studies have revealed a substantial amount of morphological variability in hybrids from a single clonal lineage [60, 61]. The diploid hybrids have been found to be at least as morphologically variable as their parental sexual species [61]. The nature of the mechanisms responsible for creating as much phenotypic variation in these asexual hybrids as in sexual species is unclear. Since the hybridizations occurred ca. 50 000 years ago [59], mutation is unlikely to explain the *C. eos-neogaeus* phenotypic variability. In the absence of interindividual genetic variation, we have hypothesized that epigenetic variation was underlying the phenotypic variability observed in *C. eos-neogaeus* hybrids. In the context of the General Purpose Genotype model, epigenetic processes could be regarded as the mechanism for extending the flexibility of their genotype.

## 3. Variation in DNA Methylation in Asexual *Chrosomus eos-neogaeus* Hybrids

We initially found that epigenetic variation was present in these fish through an MSAP survey that revealed interindividual variation in DNA methylation patterns in individuals from a single clonal lineage [47]. Importantly, the observed epigenetic variation was independent of the genotype. The hybrids came from seven geographically distant lakes characterized by different biotic and abiotic conditions. Based on their methylation profiles, individuals could be grouped according to their lake of origin [55]. The correlation observed between the environment (i.e., lake of origin) and the methylation profile strongly suggests that asexual *C. eos-neogaeus* hybrids respond to environmental variation with DNA methylation. These observations were made on one generation of organisms. We did not investigate the methylation profiles of offspring of these individuals so no conclusion can be made about the heritability of these marks.

## 4. Epigenetic Variation and Asexual Lineage Persistence

Results of previous studies and ours indicate that DNA methylation could be a viable mechanism for the creation of phenotypic variation in the studied asexual organisms, allowing them to respond to the environment in the absence of interindividual genetic variation. The presence and variation in DNA methylation have not been investigated in most asexual lineages. However, given the widespread occurrence of this modification and its presence in organisms of all the phyla where asexuals are found (except in rotifers, where the presence of DNA methylation has to our knowledge not been investigated), it is likely that many of the unstudied asexual lineages also possess DNA methylation. The ones that do not are expected to rely on other epigenetic mechanisms to regulate gene expression. For example, DNA methylation is absent in the budding yeast *Saccharomyces cerevisiae* and the fission yeast *Schizosaccharomyces pombe*. Yeast can rely on histone-modifying enzymes to control the packaging of their DNA, therefore regulating the access of their genes to transcription [64–66]. *Schizosaccharomyces pombe* also possesses RNA interference, which is notably involved in the formation of heterochromatin at their centromeres [67, 68].

Contrary to some studies where global undermethylation was observed in interspecific hybrids (e.g., [69, 70]), the methylation levels present in *C. eos-neogaeus* hybrids are comparable to those observed in other sexual vertebrates [47]. It is possible that other asexual lineages possess levels of DNA methylation comparable to those observed in *C. eos-neogaeus* and exhibit interindividual variation in their DNA methylation patterns. Through the creation of phenotypic variability necessary for facing environmental fluctuations, epigenetic processes could play a crucial role in the persistence of asexual lineages. In the next paragraphs, we will discuss the mechanisms by which some asexual lineages could be particularly apt at creating epigenetic variation among individuals and present some of the implications of epigenetic variation in asexual lineages.

## 5. Mechanisms for Variation in DNA Methylation

The capacity for phenotypic variation through epigenetic processes could explain the success of some asexual lineages. It is possible that these asexual lineages possess particularly efficient mechanisms for generating epigenetic variation.

The enzymes responsible for DNA methylation are the DNA methyltransferases (Dnmt). In mammals, where this epigenetic modification is well studied, the Dnmt3 family is responsible for *de novo* methylation: it establishes new methylation marks on previously unmethylated DNA. The Dnmt1 family of enzymes is responsible for maintenance methylation: it reestablishes the preexisting methylation pattern on the daughter strand after DNA replication. Dnmt1 prefers hemimethylated to unmethylated sites and typically maintains the methylation pattern with 95% accuracy [71]. The error rate of Dnmt1 is therefore much higher than that of DNA polymerase, making epimutations much more likely than mutations. Indeed, the number of epimutations detected in *C. eos-neogaeus* hybrids was much higher than the number of mutations [47].

A mutated copy of Dnmt1 with a decreased preference for hemimethylated DNA would lead to more errors in the propagation of the DNA methylation pattern and an increase in *de novo* methylation at previously unmethylated sites. A byproduct of this would be a greater capacity for creating epigenetic variation among asexual individuals.

Since many asexual lineages result from interspecific hybridizations, genes can be misexpressed due to mismatches between regulatory elements of the genomes of the two species [72]. For example, at a given gene, the interaction between the trans-regulatory elements of one species with the cis-regulatory elements of the other can lead to dysregulation of this gene. Through such dysregulation, asexual lineages resulting from interspecific hybridizations could show, for example, insufficient expression of Dnmt1, leading to a decreased capacity in faithfully copying DNA methylation patterns through cell divisions. Dysregulation could also disrupt the temporal expression pattern of Dnmt3: the enzyme would not only be expressed during the hybrid's development but also throughout its life. New methylation marks could then be established throughout the individual's life, greatly extending its capacity for phenotypic variation.

## 6. Epigenetics and Asexual Hybrids

When considering how asexual organisms respond to their environment, it is important to take into account that many asexual lineages result from interspecific hybridizations. Global repatterning of DNA methylation can occur upon hybridization and polyploidization. As exemplified by work in plants, methylation patterns can be radically altered [32, 73–76].

**Figure 2:**
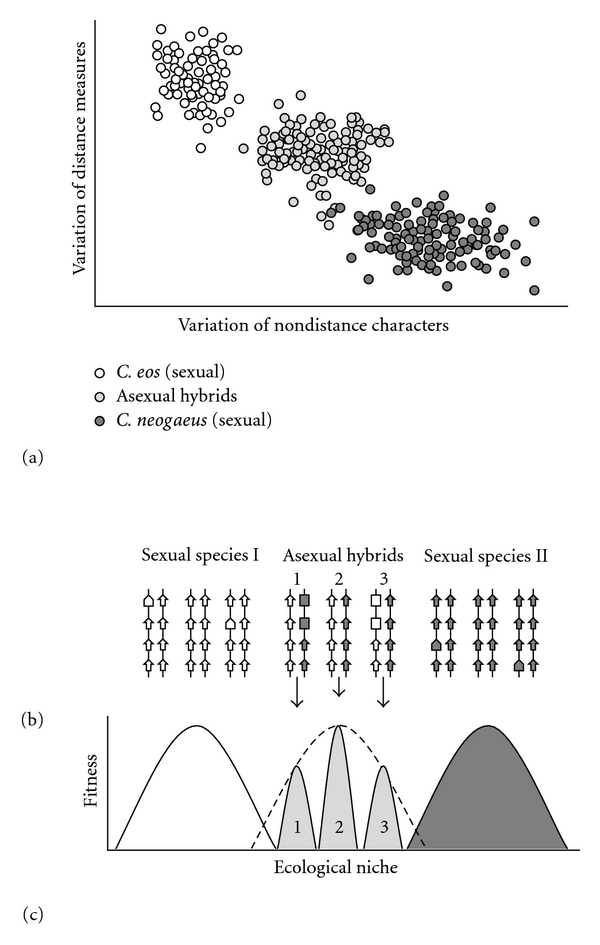
Hypothesis of the epigenetic mechanism underlying the flexibility of a genotype. (a) Phenotypic variation observed in sexual and asexual species. The points represent individual scores of *Chrosomus eos*, *C. neogaeus*, and asexual hybrids from two principal component analyses performed on body distance and nondistance measures (modified from [61]). In sexual species, the phenotypic variation among individuals is mostly the result of genetic variation, whereas, in asexual hybrids, it results from differentially expressed alleles of a same genotype. (b) Putative genetic and epigenetic variation at four genes is represented for three individuals per species. Arrows refer to expressed genes, larger arrows to different alleles of an expressed gene (genetic difference), and blocks to silenced genes (epigenetic difference). (c) Under the General Purpose Genotype model, an epigenetically flexible genotype may provide a wide ecological niche for asexual hybrids, where each different epigenetic variant would occupy a narrower niche.

Asexual hybrids might not only be able to differentially express their genes but also the specific alleles of their genes, as reported in numerous diseases where heterozygotes exhibit a diversity of symptoms according to the level of expression of the mutant allele [77–79]. *Chrosomus eos-neogaeus* hybrids could achieve this differential allelic regulation through epigenetic modifications such as DNA methylation. These hybrids possess a *C. eos* allele and a *C. neogaeus* allele for every one of their genes. For a given gene, some individuals could have a methylated *C. eos* allele and others a methylated *C. neogaeus* allele, conserving expression of the *C. neogaeus* and *C. eos *allele, respectively (Figure 2). Supposing many of their genes could be regulated this way, the number of ways in which a single genotype could be expressed would be greatly increased (theoretically 3^*n*^, where *n* is the number of genes where differential allelic expression occurs, 3 refers to expression of alleles from *C. eos* only, *C. neogaeus* only, or from both *C. eos* and *C. neogaeus*). This would greatly increase their capacity for phenotypic variation. It is unclear how this differential allelic silencing would occur, but it could be in response to an environmental cue or randomly. In *C. eos-neogaeus*, Letting et al. [80] have observed at two different genes that the *C. eos* allozyme was more expressed than the *C. neogaeus* allozyme.

Surveys of the transcriptome of *C. eos- neogaeus* hybrids have also given some preliminary evidence for differential allelic expression. Using cDNA-AFLP [81], we compared among hybrids the expression of (i) alleles common to both parental species (*C. eos-neogaeus* band found in *C. eos* and *C. neogaeus*) with that of (ii) alleles specific to one of the parental species (*C. eos-neogaeus* band found only in *C. eos* or *C. neogaeus*). In case (ii), it is possible to detect differential allelic expression whereas this is not possible in case (i) because of the dominance effect of AFLP. An absence of detection for (i) can therefore only mean that the gene is not expressed. A survey of cDNA fragments was performed on the muscle tissue of 26 genetically identical *C. eos-neogaeus* individuals. Out of 424 cDNA fragments, 75% were common to both parental species (i) while 25% were specific to one or the other parental species (ii). Interhybrid variation for the presence of these fragments was found at 10 species-specific loci (ii) (9.4%) but not at loci shared between species (i) (Fisher Exact Probability Test *P* = 0.000003) [82]. That the variation detected was only at allele-specific cDNAs suggests that, for a given tissue, differential allelic regulation among individuals could be more frequent than differential gene regulation.

As previously mentioned, it is assumed that asexual lineages will accumulate potentially deleterious mutations faster than sexual organisms because they do not possess recombination. Several studies have indeed demonstrated that asexual lineages accumulate potentially harmful mutations at a higher rate than their sexual congeners [83–85]. However, these studies did not demonstrate whether there was a phenotypic consequence to this increased mutation rate. What if it was possible to target these sequences containing mutations with DNA methylation? These potentially harmful mutations would be silenced, allowing asexuals to evade their phenotypic consequences [32, 53]. Silencing of deleterious mutations through DNA methylation could be particularly prevalent in polyploid asexuals. Many asexual lineages resulting from hybridizations are characterized by the presence of polyploids. If a polyploid organism gains a mutation in one of its gene copies, this mutation could be epigenetically silenced and the organism would still retain sufficient levels of expression through its two (or more) other copies.

These epigenetically masked mutations would represent some form of hidden genetic variation. Similarly to the evolutionary capacitance observed with Hsp90 [86], this hidden genetic variation could be exposed under certain conditions, leading to the production of new phenotypes. Such a mechanism could have allowed the accumulation of mutations in bdelloid rotifers characterized by the Meselson effect.

## 7. Heritability of Variation in DNA Methylation

The existence of environmentally induced epigenetic variation that can be transmitted to offspring poses a challenge to the modern evolutionary synthesis, which is based on the assumption that random genetic variation, impervious to environmental influences, is the only source of heritable variation in natural populations [87]. In this context, it has been argued that epigenetic variation must be heritable to be of evolutionary relevance (e.g., [33, 54]). Organisms from different taxa appear to be uneven in their capacity for transgenerational epigenetic inheritance. In mammals, methylation reprogramming in mammalian primordial germ cells is quite extensive [88, 89]. Erasure of methylation patterns also occurs in zebrafish development [90]. Therefore, it seems there is a limited potential for DNA-methylation-driven transgenerational epigenetic inheritance in vertebrates. However, this erasure is not always complete and there are a few cases of transmission across generations of variation in DNA methylation in mammals [46, 54, 91].

The extensive reprogramming in DNA methylation observed in mammals is not common to all multicellular organisms. In plants, methylation resetting in the germ line is not as extensive and examples of inheritable variation in DNA methylation are more common [46, 53, 89]. Consistently, the variation in DNA methylation detected in asexual plants by Verhoeven et al. [36, 37] was transmitted across generations.

Even though their potential for epigenetic inheritance through DNA methylation is reduced compared to that of plants, epigenetic inheritance in animals (as well as plants) could be associated with histone marks or small RNAs transmitted in the oocyte and sperm [89]. For example, transmission of phenotypic variation to offspring by nongenetic factors was detected in bdelloid rotifers [92].

As previously mentioned, we did not assess whether the environmentally associated variation in DNA methylation observed in *C*. *eos-neogaeus* hybrids could be transmitted to offspring. However, even if this variation is restricted to a single generation, it could still be relevant to the persistence of these organisms.

Heritable epigenetic variation is useful if the environment is stable across generations. Environments are however rarely completely stable, and most individuals will have to deal with environmental stresses during their lives. Epigenetic modifications, by increasing the phenotypic spectrum of a given genotype, can provide an alternative way to respond to environmental fluctuations [20]. The relevance of epigenetic mechanisms would in this case lie in their capacity to create phenotypic plasticity, not adaptation. In such cases, it is not the epigenetic mark that is transmitted across generations but the genetically encoded capacity for creating epigenetic variation that can drive phenotypic plasticity. In this case, contrary to the case where epigenetic variation is inheritable, the nature of the heritable material remains genetic, which is not in contradiction with the modern evolutionary synthesis.

In this paper, we have argued that epigenetic modifications are an important mechanism for asexual organisms to face environmental variability. We have highlighted examples in genetically identical asexual organisms where variation in DNA methylation corresponded to environmental variation. Different taxa present different susceptibilities to transgenerational epigenetic inheritance. Epigenetic modifications do not need to be inheritable to be of relevance. In fluctuating environments, it could be favorable to wipe out at least some epigenetic marks every generation. Finally, epigenetic mechanisms, though they play a crucial role in the response to environmental variation, are most likely not the only factors involved in asexual persistence. Long-term survival is likely to be due to a combination of short-term epigenetic and long-term genetic processes.
